# Recurrence and mortality according to Estrogen Receptor status for breast cancer patients undergoing conservative surgery. Ipsilateral breast tumour recurrence dynamics provides clues for tumour biology within the residual breast

**DOI:** 10.1186/1471-2407-10-656

**Published:** 2010-11-30

**Authors:** Romano Demicheli, Ilaria Ardoino, Patrizia Boracchi, Danila Coradini, Roberto Agresti, Cristina Ferraris, Massimiliano Gennaro, William JM Hrushesky, Elia Biganzoli

**Affiliations:** 1 Scientific Direction, Fondazione IRCCS Istituto Nazionale Tumori, Milano, Italy; 2 Medical Statistics and Biometry, Fondazione IRCCS Istituto Nazionale Tumori, Milano, Italy; 3 Medical Statistics and Biometry, Università di Milano, Milano, Italy; 4 Breast Surgery, Fondazione IRCCS Istituto Nazionale Tumori, Milano, Italy; 5 The University of South Carolina, Dorn VA Medical Center, Columbia, SC, USA; 6 Medical Statistics and Biometry, Università di Milano e Fondazione IRCCS Istituto Nazionale Tumori, Milano, Italy

## Abstract

**Background:**

the study was designed to determine how tumour hormone receptor status affects the subsequent pattern over time (dynamics) of breast cancer recurrence and death following conservative primary breast cancer resection.

**Methods:**

Time span from primary resection until both first recurrence and death were considered among 2825 patients undergoing conservative surgery with or without breast radiotherapy. The hazard rates for ipsilateral breast tumour recurrence (IBTR), distant metastasis (DM) and mortality throughout 10 years of follow-up were assessed.

**Results:**

DM dynamics displays the same bimodal pattern (first early peak at about 24 months, second late peak at the sixth-seventh year) for both estrogen receptor (ER) positive (P) and negative (N) tumours and for all local treatments and metastatic sites. The hazard rates for IBTR maintain the bimodal pattern for ERP and ERN tumours; however, each IBTR recurrence peak for ERP tumours is delayed in comparison to the corresponding timing of recurrence peaks for ERN tumours. Mortality dynamics is markedly different for ERP and ERN tumours with more early deaths among patients with ERN than among patients with ERP primary tumours.

**Conclusion:**

DM dynamics is not influenced by the extent of conservative primary tumour resection and is similar for both ER phenotypes across different metastatic sites, suggesting similar mechanisms for tumour development at distant sites despite apparently different microenvironments. The IBTR risk peak delay observed in ERP tumours is an exception to the common recurrence risk rhythm. This suggests that the microenvironment within the residual breast tissue may enforce more stringent constraints upon ERP breast tumour cell growth than other tissues, prolonging the latency of IBTR. This local environment is, however, apparently less constraining to ERN cells, as IBTR dynamics is similar to the corresponding recurrence dynamics among other distant tissues.

## Background

Our previous work [1] provides evidence that, when mastectomy and axillary dissection are performed as initial treatment for breast cancer, there is a very predictable nonlinear temporal pattern to the subsequent risk for recurrence and death. There are two peaks in recurrence risk, regardless of the hormone receptor status of the resected primary tumour. The first occurs very early, at the second-third year, while the second peak occurs years later. Even though the risk peaks occur at about the same time following primary cancer resection, regardless of tumour hormone receptor status, the overall risk level of early recurrence is much higher for patients bearing Estrogen Receptor (ER) negative (N) tumours than for those bearing ER positive (P) tumours. The second later recurrence peak is, oppositely, higher for ERP and lower for ERN tumours. Thus the two hazard curves intersect at the third year, as observed by others [2,3]. Contrary to recurrence dynamics, however, the hazard rate pattern for mortality displays simpler dynamics, as death for ERP tumours is delayed compared to ERN tumours.

The distinct and predictable recurrence and subsequent death risk patterns indicate a likely synchronizing effect of the surgical resection upon subsequent metastatic cancer development, apparently similar in all seeded distant organs [4]. This synchronizing effect, probably accelerates early recurrence and subsequent death, occurs for both types of breast cancer but much more lethal in the early post surgery span for ERN tumours. The observed differences in the mortality risk pattern between ERP and ERN tumours may in part be explained by the higher frequency of visceral metastasis for ERN tumours [5,6], and also by their lack of response to hormone therapy [7,8]. Additionally, the resection-associated recurrence following primary tumour resection displays an apparently similar time-course for every ER class, suggesting caution in the interpretation of the clinical meaning of the two main subtypes of breast cancer identified by epidemiological features linked to ER expression [9] and gene expression profiling (luminal A and basal-like, phenotypically referred as ERP and ERN, respectively) [10] as well as the distinct gene expression patterns associated with ER status [11].

Our earlier findings [1] were described in an era of larger primary resections. Since breast cancer surgery has become progressively more conservative, we now report whether the same bimodal breast cancer recurrence patterns follow smaller operations. The availability of ten year follow up for nearly three thousand such patients treated at the Milan National Cancer Institute allows us to determine whether the dynamics of recurrence following a lesser resection has the same shape over time as mastectomy; whether it is affected by the difference between tumourectomy and the larger conservative resection of quadrantectomy; whether the dynamics of distant recurrence differ by ER status of the primary tumour in this surgical setting and whether the dynamics of post resection differ according to the organ site of that distant spread. Finally, because of the large number of patients studied we are also able to determine whether these recurrence dynamics differed when the recurrence occurred within the ipsilateral previously resected breast as compared to other distant sites.

## Methods

The data derived from patients undergoing conservative surgery within a series of randomized clinical trials carried out at the Milan National Cancer Institute, investigating the role of different approaches for the primary tumour treatment, were scrutinized. In the Milan Institute, since the preliminary results of the first trial on the conservative treatment of early breast cancer [12] were reported to be as good as or better than more aggressive resections, this treatment became routine practice. This has allowed for data from cases comparably treated outside randomized clinical trials (out-trial patients) to be systematically recorded as for in-trial patients and this database was included in the analysis as well.

In one trial, patients with invasive breast carcinoma 2,5 cm or less were randomized to either quadrantectomy, complete axillary dissection and radiotherapy (QuaRT) (360 women) or tumourectomy plus axillary dissection and radiotherapy (TaRT) (345 women). Quadrantectomy involved radial breast resection with excision of 2-3 cm of normal tissue around the tumour plus the removal of a sufficiently large portion of overlying skin and underlying fascia whilst lumpectomy removed only the tumour mass with a margin of normal tissue of 1 cm. In a succeeding trial eligibility criteria were identical and patients were randomized to QuaRT (294 women) or quadrantectomy plus axillary dissection without radiotherapy (Quad) (273 women). The full series of out-trial patients, who received QuaRT for unilateral primary breast cancer, amounts to 1652 patients. All analyzed trials were performed with the approval of the ethics committee of the Milan National Cancer Institute and patients were enrolled following informed consent.

With the exception of patients allocated to the Quad arm, who did not receive radiotherapy, all patients received a radiation dose of 60 Gy to the ipsilateral breast. All axillary node positive (N+) patients were offered systemic adjuvant treatment [Cyclophosphamide plus Methotrexate plus Fluorouracil (CMF) or CMF plus Doxorubicin (Dx)], while no further post-surgical systemic treatment was recommended to axillary node negative (N-) patients. Adjuvant hormone therapy was not utilized within the randomized clinical trials and infrequently employed for out-trial patients, as it was not considered mandatory at that time. Details of the two trials and of the out-trial series have been reported elsewhere [13-15].

All baseline data, treatment features and relevant clinical events were collected in standard format and stored in a clinical database, from which, after excluding 99 patients for whom the pathological T classification could not be determined, data of the 2552 patients undergoing conservative surgery plus RT and the 273 patients receiving quadrantectomy without RT were extracted for the present analysis. ER status was measured by the dextran-coated charcoal method and tumours with ER values >10 fmol/mg protein were considered ERP.

Ipsilateral breast cancer recurrence (IBTR) and distant metastasis (DM) as first events and death from any cause were considered in the analysis. IBTR was defined as any new breast cancer focus appearing in the operated breast. DM was defined as any breast cancer manifestation(s) in areas other than that of IBTR with the exception of the contralateral breast. All diagnosed primary tumours, including contralateral breast cancers, were considered as competing events. In order to avoid the usual uncertainties related to the cause of death, deaths from any cause were studied instead of breast cancer related deaths only.

Flexible piecewise exponential regression models for the hazard function were performed by subdividing observed time data in three months intervals [1]. For a smoothed estimate of the hazard function, Natural Cubic Spline with fixed boundary knots, at which the natural (linear) boundary conditions are imposed, were used for their better conditioning on the tail in avoiding fluctuations due to few and strewn events on late follow-up. These enforce the constraint that the function is linear beyond boundary knots. Boundary knots were imposed in 1.5 - first observed time as default - and in 106.5 - as no more than a dozen events are left on the right side. Models included interaction terms, allowing for the estimate of the possible change of the hazard according to ER status. The evidence of different behaviours was informally assessed by model selection according to the Akaike Information Criterion (AIC). When studying hazard rates according to ER status following an exploratory perspective, models with a different shape for the hazard according to ER status were adopted. Since the response of the used regression model is the logarithm of the cause-specific hazard, the 95% confidence interval is based on log-transformation and this fact prevent them to be symmetric. The width of confidence intervals are mainly determined by the sample size and the size of the uncensored subject group.

Crude cumulative incidence for IBTR and DM was non-parametrically estimated to account for the presence of competing events.

## Results

### Patient Characteristics

The main characteristics of patients are reported in Table 1. Median age was 49 years (range 21 - 79 years) and 57.1% of patients were premenopausal. Median tumour size was 1.5 cm (range 0.2 - 4.3 cm) and 38.2% of cases had axillary lymph node invasion. Adjuvant systemic chemotherapy (CMF ± Dx) was administered to 73.2% of N+ patients, and 153 patients (14.2%) received Tamoxifen only.

**Table 1:** Main patient characteristics

	*All*	*ERP*	*ERN*
Total number	2825	1811	482
Age			
≤ 45	972	551	184
46 - 55	992	643	179
56 - 65	631	453	89
>65	230	164	30
Menopausal status			
Pre	1613	972	291
Post	1201	837	190
Unknown	11	3	1
Tumor size			
≤ 2 cm	2428	1558	383
> 2 cm	397	253	99
Nodal status			
N-	1745	1097	299
N + (1-3)	771	504	122
N + (>3)	309	210	61
Adjuvant therapy (for N + patients)			
none	68	39	12
CMF ± Dx	790	513	145
Tamoxifen	153	120	17
Other	10	5	
Unknown	59	37	9

The median follow-up time is 121 months. The number of recurring patients and the number of deceased patients at 5 and 10 years of follow-up are reported in Table 2. Hormone receptor status was obtained for 81.2% of tumours (Table 1). Patients with ERN tumours were more likely premenopausal and had tumours slightly larger than patients with ERP tumours, while the axillary nodal status was similar in both ER levels.

**Table 2:** Number of events at 5 and 10 years of follow-up

	5 years	10 years
Cons. Plus RT - 2552 patients		
IBTR	130	217
DM	396	521
Deaths	478	590
Quad - 273 patients		
IBTR	41	62
DM	22	37
Deaths	47	66

### Overall Outcomes

At 10 years of follow-up, ERP and ERN patients showed the same crude cumulative incidence of total recurrence (36%) as well as similar frequency of IBTR (12.6% and 11.1%, respectively) or DM (23.9% and 24.6%, respectively); suggesting that ER status *per se *did not influence the cumulative rate of recurrence. Also, the late outcome for ERP and ERN tumours was similar with global survival of 76.6% and 72.7% respectively.

### Distant Recurrence Dynamics

**Figure 1:**
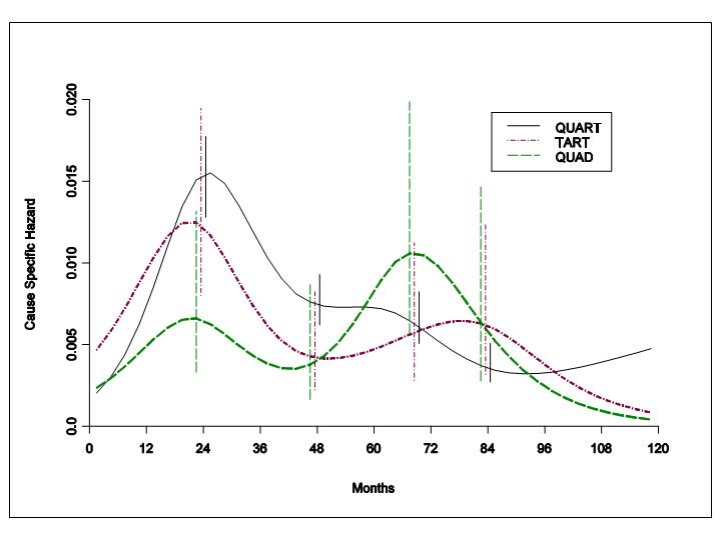
**Hazard rate estimates for distant metastasis in 2204 patients undergoing QuaRT, 348 patients receiving TaRT and 273 patients given Quad**. The ubiquitous bimodal distant recurrence dynamics described previously following mastectomy are present when much smaller operations are employed. Radiation of the chest wall clearly does not eliminate or even delay the first early recurrence peak. Vertical lines represent point-wise confidence interval for the model estimated hazards, according to standard asymptotic theory.

The patterns of the hazard rate for DM for patients undergoing different local treatments are reported in Figure 1. The bimodal recurrence dynamics (early peak at about 24 months, late peak at the sixth-seventh year) is manifest for all treatment groups.

**Figure 2:**
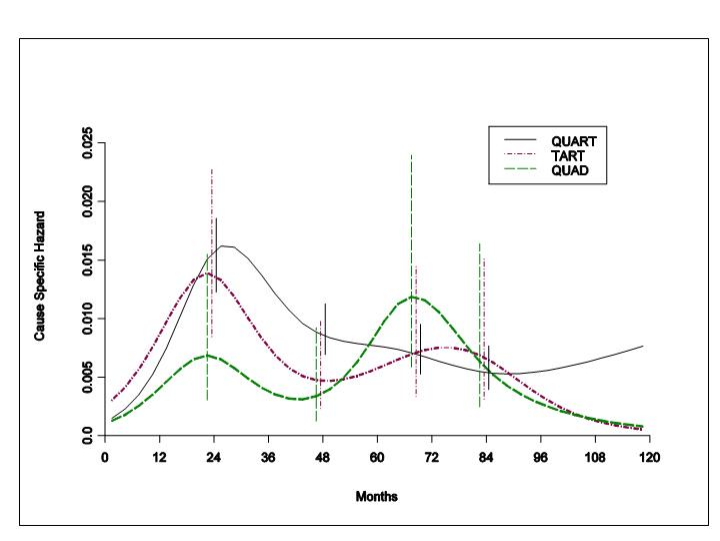
**Hazard rate estimates for distant metastasis in patients with ERP tumours undergoing QuaRT (1354 patients), TaRT (261 patients) and Quad (196 patients)**. There is virtual identity of distant recurrence dynamics for each conservative local treatment modality among ERP tumours. Vertical lines represent point-wise confidence interval for the model estimated hazards, according to standard asymptotic theory.

**Figure 3:**
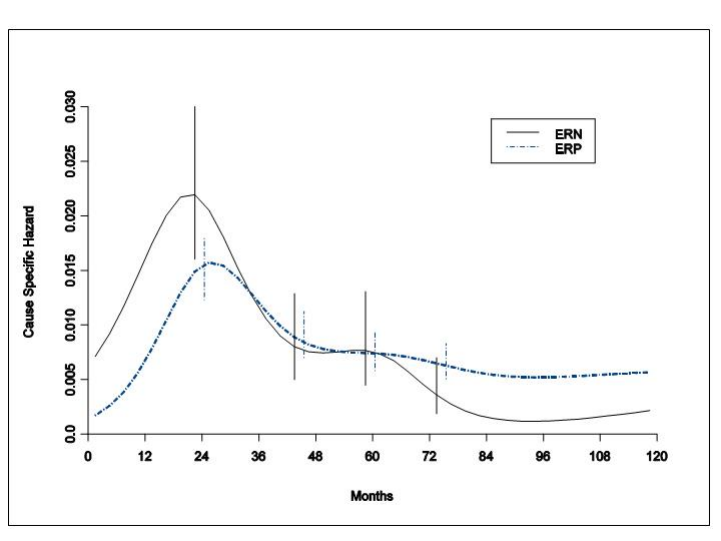
**Hazard rate estimates for distant metastasis in 1615 ERP tumours 427 ERN tumours from patients undergoing conservative surgery (quadrantectomy or tumourectomy) plus RT**. These data show that the recurrence peak timing after conservative resection is maintained regardless of tumour hormonal receptor status. Patients with ERN tumours do worse during early years and better later. Vertical lines represent point-wise confidence interval for the model estimated hazards, according to standard asymptotic theory.

When the DM dynamics was related to the ER status, patients with ERP tumours receiving different local treatments displayed hazard rates for DM with remarkably similar patterns (Figure 2). Regrettably, similar comparison could not be performed for patients with ERN tumours, because of inadequate number of such patients receiving TaRT or Quad. Therefore, the recurrence risk patterns by ER status were estimated and compared within the group of patients receiving conservative surgery (quadrantectomy or tumourectomy) plus RT. Once again a bimodal structure emerged for both ER levels with a first dominant early peak at the end of the second year after primary tumour removal (Figure 3). During the third year the two curves intersect and the DM risk for ERN tumours drops under the corresponding value of ERP tumours after the fifth year.

**Figure 4:**
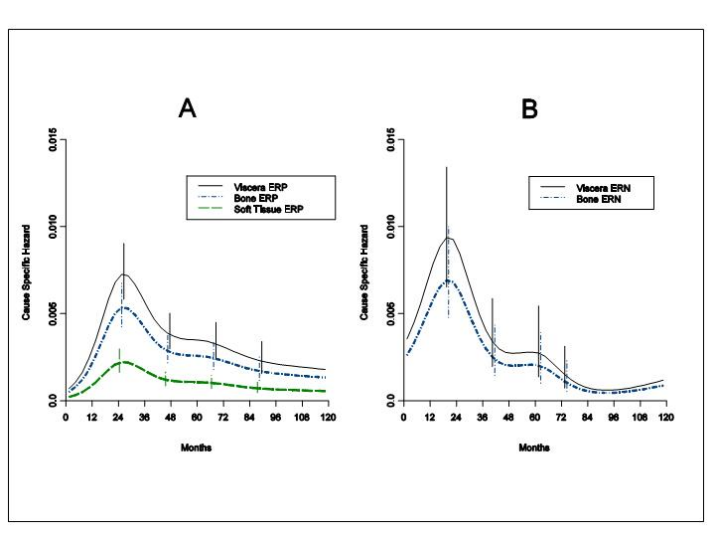
**Hazard rate estimates for distant metastasis by metastatic site in 1528 ERP tumours (A) and in 391 ERN tumours (B) from patients undergoing conservative surgery (quadrantectomy or tumourectomy) plus RT**. The similarity of recurrence dynamics provides evidence that recurrence timing is generated by factors influencing the metastatic development, regardless of the seeded organ. Vertical lines represent point-wise confidence interval for the model estimated hazards, according to standard asymptotic theory.

Finally, the DM dynamics in different metastasis sites (soft tissue, bone, viscera) was analyzed in the subset of patients for whom the treatment protocol required recording this information. Such patients received conservative surgery plus RT. The model allowing for the cause specific hazard to have a different behaviour according to ER group, but only a different scale level according to the different metastatic sites, was actually selected in agreement with previous results [4]. For ERP tumours the hazard rate curves display similar bimodal pattern for all sites, albeit with different risk levels (Figure 4A). Comparable results emerge for DM to viscera and bone for patients with ERN tumours, while the limited number of events prevented to reliably analyze DM to soft tissue for these patients (Figure 4B).

### Local Recurrence Dynamics

**Figure 5:**
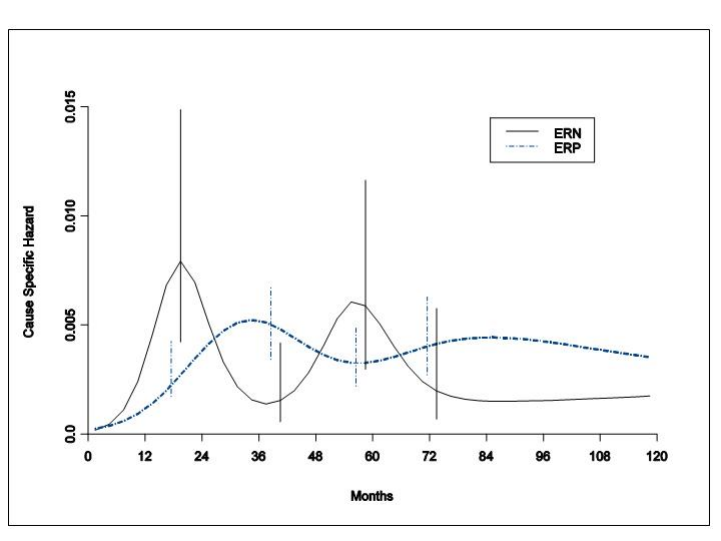
**Hazard rate estimates for IBTR in 1615 ERP tumours and in 427 ERN tumours from patients undergoing conservative surgery (quadrantectomy or tumourectomy) plus RT**. The dynamics of recurrence within the same breast irradiated following tumourectomy or quadrantectomy is quite distinct and different depending upon whether the resected tumor does or does not bear sex hormone receptors. Vertical lines represent point-wise confidence interval for the model estimated hazards, according to standard asymptotic theory.

IBTR dynamics was analyzed in patients undergoing conservative surgery plus RT. The general bimodal pattern emerges for both ER levels (Figure 5). However, the dynamics of local recurrence are quite different depending upon whether the resected tumor does or does not bear sex hormone receptors. Hormone receptor negative cancers recur in the resected irradiated breast with a sharp high early peak and with a clear second peak four years later. Hormone receptor bearing primary tumors recur with a broader and lower first peak and a wide later peak. Both the early and the late peak of ERP tumours are delayed of about 1,5 and 2,5 years, respectively, in comparison with the corresponding peaks of ERN tumours. The slight overlapping of the confidence intervals does not conflict with the major hazard rate patterns, although some major care is needed for later times when events are observed to be quite spread.

### Mortality dynamics

**Figure 6:**
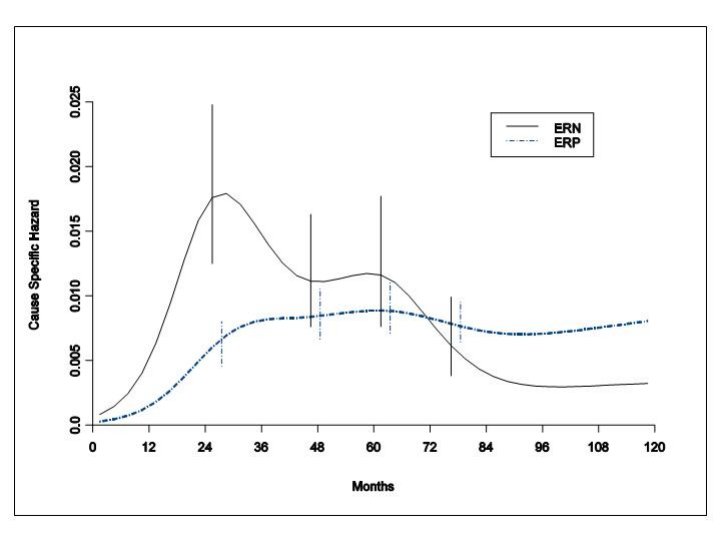
**Hazard rate estimates for mortality in 1615 ERP tumours and 427 ERN tumours from patients undergoing conservative surgery (quadrantectomy or tumourectomy) plus RT**. The mortality risk patterns reveal differences between ERP and ERN tumours in the clinical course of the disease during the critical period spanning from clinical recurrence to death. Vertical lines represent point-wise confidence interval for the model estimated hazards, according to standard asymptotic theory.

In spite of the fact that the cumulative mortality is quite similar for both ER categories, the mortality chronology is definitely different for ERP and ERN tumours (Figure 6). For patients with ERP tumours, the mortality risk includes a slow increase in level reaching a plateau at the fifth-sixth year, while for patients with ERN tumours the rise is much steeper with a spike at the third year. ERN tumours show a further mortality peak at about 60 months while for ERP tumours a second mortality peak is blurry indistinct and hardly detectable at the same time. The hazard rate curve of patients with ERN tumours crosses the corresponding curve of patients with ERP tumours between the sixth and the seventh year and persists at a lower level afterwards.

## Discussion

### Distant Recurrence Dynamics

This explorative investigation of a large and mature database provides evidence that the bimodal DM dynamics, previously observed in patients undergoing mastectomy for early breast cancer, is also present in patients undergoing more conservative resections with or without RT. The peak recurrence timing for these patients (Figure 1) is comparable to that observed in patients undergoing mastectomy [1,16]. These data further support our hypothesis that surgical synchronization of distant recurrence occurs even after smaller operations. This supports the concept that local treatment modalities do not appreciably modify the growth traits of the DM process. In Figure 1, the early risk level for patients not given RT is considerably lower than the corresponding risk of patients receiving RT. This counterintuitive finding may be reasonably related to the fact that the omission of RT over the resected breast area results in very high rate of IBTR as first event that may in turn partially obscure the competing DM rate. In spite of this, the DM risk pattern for these patients emerges unmistakably, confirming that RT to the chest wall does not eliminate or even delay the first early recurrence peak. In a similar way one can explain the finding that patients receiving TaRT show a moderately lower first peak height than patients given QuaRT, who experienced lower IBTR rates, as it was already noted [13].

The similarity between the hazard rate patterns for all patients and for patients with ERP tumours (Figures 1 and 2), indirectly suggests that ER status does not appreciably change the DM dynamics for any local treatment modality in this subset of patients most of whom did not receive hormone therapy. This notion is further supported by the analysis of the DM risk pattern by ER status for patients receiving postoperative RT (Figure 3). Both ERP and ERN tumours display similar bimodal dynamics, regardless of the small differences in a few prognostic factors between the two groups. The two peaks, however, have different ER-related heights with the intersection of the two curves at three years, a result that is nearly superimposable to what was obtained for patients undergoing mastectomy [1].

An additional result supporting the lack of relevance of the local treatment on the metastatic process is provided by the analysis of DM dynamics by site (Figure 4) that yields results substantially identical to what was obtained for patients undergoing mastectomy [4].

### Local Recurrence Dynamics

Although the analysis of the IBTR dynamics yields a bimodal hazard rate pattern for both ERP and ERN tumours, there is a profound difference in the peak timing between the two ER categories (Figure 5). This finding is a remarkable exception to the recurrence risk rhythm, thoroughly congruent with a common metastasis development pathway, till now observed in all analyzed subsets (by tumour size, nodal status, menopausal status, ER content, recurrence site) of patients undergoing radical and conservative surgery with or without adjuvant chemotherapy [1,4,16]. This timing signal difference may be a clue to different biological mechanisms responsible for the single complex clinical event that conventionally is ascribed to the outgrowth of residual foci of malignant cells.

At least three mechanisms could be involved in IBTR origin. A) Over 60% of all primary breast cancer specimens contain additional foci of in-situ or micro-invasive disease most of which are outside the index quadrant [17,18], representing latent disease biologically similar to a new primary. B) The tumour bed is fertile soil with the capacity for reseeding of circulating cancer cells, with the IBTR occurring very much like the natural history of loco-regional recurrence after mastectomy (1). C) Microscopic foci of residual disease may remain in the tumour bed following surgery whereby the cytokine soup in the local environment might kick start outgrowth cancer resulting in an early peak of IBTR. The second and third mechanism might explain a selective advantage for ERP cancer cells to re-grow or reseed in this zone with a different dynamic to that of the ERN cancer cells. Amongst other unique local features the adipose tissue is rich in the enzyme aromatase that is responsible for the peripheral conversion of circulating androgens from the adrenal gland into oestradiol [19]. For IBTR dynamics, however, no timing effect is expected by the first mechanism, while the two others would theoretically shift peaks of ERP tumours in an opposite direction in comparison with findings.

IBTR may also be produced by tumour cells lodged within the breast to which adjuvant RT is administered. RT causes both a conventional short-term tumouricidal effect and the so called "tumour bed effect", in which the radiation-induced vascular damage of the surrounding tissue more indirectly impairs tumour growth [20]. We cannot, however, rule out that the different recurrence peak timing is related to a differential effect of RT on ERP and ERN tumour cells. This hypothesis is, however, weakened by the fact that in experimental models ERN tumour cells display higher radiosensitivity than ERP tumour cells [21], thus suggesting a possible retarding effect contrary to the observed one.

Summarizing, different mechanisms could account for IBTR timing namely: a mechanism identical to a new primary, a mechanism equivalent to metastatic re-seeding, a mechanism related to the outgrowth of residual disease (unique for each hormone receptor phenotype) and finally the tumour bed effect. None of them seems to yield a persuasive explanation. Therefore, we favour a more general hypothesis, namely that the ERP status *per se *is related to delayed IBTR appearance.

To colonize a new organ, disseminated tumour cells must have the capacity to effectively interact with the new microenvironment [22], both during a phase of tumour dormancy and while active growth is going on [16]. Different organ microenvironments may impose distinct requirements for complete clinically relevant colonization, thus resulting in a selective metastasis distribution [23]. In spite of these constraints, implying heterogeneity of the seeded organ related conditions, the hazard rate for recurrence in different sites display a remarkably uniform pattern [1,4,16] (that is confirmed in the present analysis on DM), strongly suggesting similarity of tumour-microenvironment dynamic interactions. The uniqueness of IBTR hazard rate pattern, therefore, may be ascribed to the unique traits of the tumour-microenvironment relationship within the organ of tumour origin that apparently imposes more restrictive conditions to ERP tumour cells.

This picture is not unreasonable. Besides reports revealing the role of activated stroma on promoting tumour growth [24-26], several lines of evidence using both in vitro and in vivo model systems provide support for a suppressive role of certain types of stroma. Breast carcinoma cells exhibited a reduction in proliferation when cultured in presence of normal breast mesenchymal cells [27]. Differentiation of transformed cells was obtained in vitro in colon adenocarcinoma cell lines co-cultured with embryonic mesenchyme [28]. A suppressive effect of normal fibroblasts was observed with ras-infected epithelial cells [29] and with prostatic adenocarcinoma [30]. Normal fibroblasts inhibited tumorigenic outgrowth of normal breast epithelium in contrast to tumour fibroblasts [31]. Embryonal carcinoma cells were capable of full differentiation and co-operated to the development of normal mice upon injection into developing blastocysts [32,33]. Of note, in a three-dimensional model of human breast stromal-epithelial cell interaction, it was observed that normal breast fibroblasts (obtained by normal reduction mammoplasty), but not fibroblasts from other sites could inhibit or retard morphological transformation of normal and pre-neoplastic epithelial cell lines [34]. Moreover, and most important, normal fibroblasts had the ability to suppress oestrogen-induced growth of ERP pre-neoplastic human breast epithelial cells. It is conceivable, therefore, that the microenvironment within the residual breast tissue may play a dominant regulatory role upon ERP breast tumour cells enforcing more stringent constraints upon growth within the operated breast than other tissues, thus prolonging the latency to IBTR. By contrast, the breast tissue conditions are apparently less constraining to ERN cells, as IBTR dynamics is similar to the corresponding recurrence dynamics among other tissues.

### Mortality dynamics

The hazard rate curves for mortality reveal differences between ERP and ERN tumours in the clinical course of the disease during the critical span from clinical recurrence to death. As suggested for a similar delay observed in patients undergoing mastectomy [1], both the different frequency of recurrence in sites bearing dissimilar prognosis (viscera vs bone vs soft tissue) [5,6] and differential responsiveness to the post-recurrence administered endocrine therapy [7,8] may account for a better post recurrence prognosis for patients with ERP tumours.

### Potential selection bias

It should be acknowledged that, for analyses like the present one, there may be potential selection bias in the data. For example, there may have been differences in surgical practice or systemic therapy, since the patients were enrolled during a time span of about a decade. It is to be noticed, however, that surgery, RT and systemic treatments were performed according to constant guide-lines by the same therapy units throughout the accrual of all examined patients. Moreover, as detailed in the section "Patients and methods", the eligibility criteria of all examined series were virtually identical. In our opinion, therefore, the potential selection bias related to merging consecutive trials, which could account for differences in clinical outcome, particularly in local recurrence, is quite small.

## Conclusions

If the results of the present study are taken together with the results of similar analyses on patients undergoing mastectomy [1,4,16], it may be concluded that the DM dynamics is essentially equivalent for all tumours and is unaffected by different primary tumour removal modalities (mastectomy or quadrantectomy with or without RT or tumourectomy with RT), in agreement with the paradigm explicitly proposed by B. Fisher [35].

Local breast cancer recurrence dynamics are similar to DM dynamics for both ERP and ERN tumours following mastectomy while following conservative surgery plus RT such a similarity is restricted to ERN tumours only. Therefore, delays observed in the IBTR risk peaks for ERP tumours following conservative surgery and postoperative RT are a remarkable exception to the otherwise common recurrence risk rhythm. This clinical behaviour is likely to reveal a ER related relationship between tumour cells and the breast tissue, which may play a dominant regulatory role upon ERP breast tumour cells with more stringent constraints than upon ERN cells, for which the time to overt recurrence is similar to the corresponding time of the other tissues.

All observed regular patterns in the clinical course of a disease that conversely shows a high degree of heterogeneity modulated by host vs tumour interactions should guide translating into clinical behaviours findings from investigations on prognostic factors (e.g. studies on tumour gene expression profiles). More in general, since life is by its very nature, a terminal condition, we believe and our data support that more careful attention should be paid to the temporal patterns of cancer recurrence and death over the span following diagnosis and therapeutic intervention. Being or becoming a member of a subgroup whose death is more likely to occur very early or much later is important for the discovery and testing of therapies designed to delay these events, as well as, for planning one's life, in general. Timing is, after all, everything in all such human events.

## Abbreviations

ER: estrogen receptor; ERP: estrogen receptor positive; ERN: estrogen receptor negative; RT: radiotherapy; QuaRT: quadrantectomy plus radiotherapy; TaRT: tumourectomy plus radiotherapy; Quad: quadrantectomy; CMF: Cyclophosphamide plus Methotrexate plus Fluorouracil; Dx: Doxorubicin; IBTR: ipsilateral breast tumour recurrence; DM: distant metastasis; AIC: Akaike Information Criterion.

## Competing interests

The authors declare that they have no competing interests.

## Authors' contributions

RD: conception, design, analysis, interpretation of data, manuscript drafting, manuscript revising, final approval. IA: analysis of data, manuscript drafting, manuscript revising, final approval. PB: analysis of data, manuscript revising, final approval. DC: acquisition, analysis of data, manuscript revising, final approval. RA: acquisition of data, manuscript revising, final approval. FC: acquisition of data, manuscript revising, final approval. MG: acquisition of data, manuscript revising, final approval. WJMH: interpretation of data, manuscript drafting, manuscript revising, final approval. EB: analysis, interpretation of data, manuscript drafting, manuscript revising, final approval.

## Pre-publication history

The pre-publication history for this paper can be accessed here:

http://www.biomedcentral.com/1471-2407/10/656/prepub
